# Investigating interspecific mating in the thelytokous predatory mite *Amblyseius herbicolus* (Chant) (Acari: Phytoseiidae), with comparative observations from three sexually reproducing phytoseiid species

**DOI:** 10.1007/s10493-025-01034-6

**Published:** 2025-06-03

**Authors:** Keshi Zhang, Junlin Cao, Xintong Li, Zhi-Qiang Zhang

**Affiliations:** 1 School of Biological Sciences, University of Auckland, Auckland, 1072 New Zealand; 2 Manaaki Whenua– Landcare Research, 231 Morrin Road, Auckland, 1072 New Zealand

**Keywords:** *Amblydromalus limonicus*, *Amblyseius herbicolus*, *Amblyseius lentiginosus*, Behaviour, Copulation, *Neoseiulus Cucumeris*

## Abstract

Asexual reproduction can be advantageous in the short term but is generally considered an evolutionary dead end due to the lack of genetic diversity and the accumulation of deleterious mutations. The thelytokous predatory mite *Amblyseius herbicolus* (Chant) (Acari: Mesostigmata) shows potential for biological pest control, but its reproductive mechanism remains poorly understood. This study examined whether *A. herbicolus* females have retained mating ability by exposing them to morphologically similar heterospecific males from three sexually reproducing phytoseiid species: *Amblydromalus limonicus* (Garman & McGregor), *Amblyseius lentiginosus* Denmark & Schicha, and *Neoseiulus cucumeris* (Oudemans). No mating or insemination occurred between *A. herbicolus* females and heterospecific males within 24 h. In contrast, nearly all males successfully inseminated conspecific females in a follow-up observation. Mating behaviours varied across species, with *A. lentiginosus* showing the longest pre-mating duration and lowest insemination rate, indicating higher mate selectivity. Most *Ad. limonicus* males inseminated a single spermatophore, while *A. lentiginosus* and *N. cucumeris* deposited two spermatophores, suggesting differences in sperm allocation and competition. No correlation was found between male body size, mating duration, and endospermatophore volume, possibly due to the small sample size. This study does not establish whether *A. herbicolus* reproduces strictly through thelytoky, nor does it exclude the possibility of sexually reproducing populations elsewhere. Further research, including interspecific mating trials with the more morphologically similar *Amblyseius largoensis* and an investigation into the role of endosymbionts such as *Wolbachia*, is needed to reveal the mechanisms underlying asexuality *in A. herbicolus*.

**Supplementary Information:**

The online version contains supplementary material available at 10.1007/s10493-025-01034-6.

## Introduction

The family Phytoseiidae (Acari: Mesostigmata) contains primarily free-living, plant-inhabiting predatory mites, many of which are essential biological control agents against agricultural pests (McMurtry et al. 2015; Zhang 2003). Species such as *Amblyseius swirskii*, *Neoseiulus cucumeris* (Oudemans), and *Phytoseiulus persimilis* have been extensively utilised in pest management (Amano and Chant 1977; McMurtry et al. 2013; Zhang 2003). Maintaining a stable population of phytoseiid predators is crucial for effective pest management (Amano and Chant 1978; Xie et al. 2018; Xu et al. 2024). Consequently, their reproductive biology has been a key focus of research.

Although phytoseiid species are small (~ 300–450 μm in body length) and difficult to observe without proper tools such as a microscope, their reproductive behaviours have been well documented in various species (e.g., Amano and Chant 1979; Elbadry and Elbenhawy 1968; Hoy and Cave 1985; Pappas et al. 2005; Tsunoda 1994). Most phytoseiid species reproduce via pseudo-arrhenotoky, in which mating is required for oviposition (Hoy 1985; Norton et al. 1993). In this reproductive system females are diploid whereas haploid males arise through paternal genome elimination. Phytoseiid females possess a distinctive ‘phytoseioid-type’ sperm-access system that is unique within the order Mesostigmata (Alberti 2002; Moraza and Linquist 2011).

Unlike species with elaborate courtship displays, such as the peacock spider (*Maratus volans*) (Girard et al. 2011) or mites of the family Trombidiidae (Zhang 1999), phytoseiids exhibit relatively simple, yet diverse, mating behaviours (Elbadry and Elbenhawy 1968). Phytoseiid males are generally more active in mate searching than females (Amano and Chant 1979; Tsunoda 1994). During mating the male engages in a series of behaviours around and on the female before insemination, while the female remains relatively stationary. Phytoseiid mating behaviour has been classified into two types: ‘*Amblyseius*-*Typhlodromus*’ and ‘*Phytoseiulus*’ (Amano and Chant 1979). In the former, the male climbs onto the female’s dorsum after initial contact before moving underneath to assume a ‘venter-to-venter’ mating posture. In the latter, males directly invert themselves into this mating posture. In this posture males start to transfer sac-like spermatophores into the female’s sperm-access system via their spermatodactyl, a specialised structure on the moveable digits of phytoseiid males (Schulten 1985; Ullah et al. 2017a). Sperm is stored in two spermathecae, each receiving sperm through insemination pores located between the bases of the third and fourth legs. While a single mating event is sufficient for maximum lifetime reproductive success in some phytoseiid species, others require multiple inseminations (Amano and Chant 1978; Momen 1997; Schulten et al. 1978; Tsunoda and Amano 2001).

Most phytoseiid mites reproduce sexually, but at least 10 species reproduce asexually via thelytokous parthenogenesis (thelytoky) (Norton et al. 1993; Zhang 2022). However, males have been reported in some populations of thelytokous species such as *Amblyseius elongatus* (Oliver 1971) and *Neoseiulus tunus* (Cavalcante et al. 2017). Unlike pseudo-arrhenotoky, thelytoky enables female offspring to develop from unfertilised eggs laid by virgin females (Norton et al. 1993). The phytoseiid predator *Amblyseius herbicolus* (Chant) is one such thelytokous species, which is widely distributed and considered a promising biological control agent (Cavalcante et al. 2015; Kalile et al. 2021; Lam et al. 2021; Reis et al. 2007; Xin and Zhang 2021). Field collections and laboratory rearing have not identified males of *A. herbicolus* in New Zealand (Fan et al. 2024; Lam et al. 2021; Liu et al. 2023, 2024c; Ma et al. 2024; Zhang and Zhang 2021, 2022a, b, c, 2023; Zhang et al. 2024). Moreover, taxonomic studies of *A. herbicolus* have not reported the presence of males worldwide, except for a single study that documented their collection but did not provide a morphological description (Fang et al. 2022; Zhang 2022). Although some biological studies have suggested the presence of *A. herbicolus* males (Hou et al. 2022; Notghi Moghadam et al. 2010), these reports either misidentified the specimens or did not provide clear identification criteria.

Interspecific mating, particularly between morphologically similar species, can occur among mites (Reyer 2008; Ullah et al. 2017b). Some phytoseiid females accept interspecific matings, as observed in *Neoseiulus womersleyi* and *Neoseiulus longispinosus*, as well as in the spider mites *Tetranychus evansi* and *Tetranychus urticae* (Sato et al. 2016; Ullah et al. 2017b). However, these matings generally result in no or sterile offspring. Although *A. herbicolus* females possess spermathecae, it remains unclear whether they can behaviourally accept mating and be inseminated by males of closely related species. In an attempt to clarify this, we investigated interspecific matings between *A. herbicolus* females and males of three phytoseiid species: *Amblydromalus limonicus* (Garman & McGregor), *Amblyseius lentiginosus* Denmark & Schicha, and *N. cucumeris*. These four species vary in phylogenetic relatedness yet share a type-III generalist predator lifestyle by preying on various insect and mite species and utilising plant products such as pollen and nectar (McMurtry et al. 2013; Wang et al. 2024).

We hypothesised that *A. herbicolus* females would mate with and be inseminated by *A. lentiginosus* males, given their relatively close phylogenetic relationship (i.e., of the same genus) and morphological similarities, but not with the more distantly related *Ad. limonicus* or *N. cucumeris*. This study aimed to provide insights into the origin of the mode of reproduction in *A. herbicolus* and assess whether females of this species retain the ability to mate.

To confirm male viability of *Ad. limonicus*, *A. lentiginosus*, and *N. cucumeris*, we paired them with conspecific females and observed their mating behaviour. To our knowledge the mating behaviours of *Ad. limonicus*, *A. lentiginosus*, and *N. cucumeris* have not been previously reported. We hypothesised that their mating behaviours would be consistent due to their similar lifestyles. This study also aimed to enhance our understanding of mating behaviour and reproductive strategies in phytoseiid mites.

## Materials and methods

### Mite cultures

All mites used in this study were obtained within New Zealand and maintained under controlled laboratory conditions at Manaaki Whenua– Landcare Research (St Johns, Auckland, New Zealand) (see Liu et al. 2024b for details of the mites). The dried fruit mite, *Carpoglyphus lactis* (L.) (Acari: Carpoglyphidae), was used as prey to feed all examined phytoseiid predators. *C. lactis* was cultured on a Petri dish containing a mixture of wheat bran (~ 90%) (Goodman Fielder Limited, New Zealand), sugar (~ 5%) (Chelsea, New Zealand), and dry yeast (~ 5%) (Goodman Fielder Limited, New Zealand), all sourced locally.

To maintain the cultures, a rearing set-up was established using a plastic container filled with water. A sponge was placed inside the container, supporting a black plastic sheet, on which the Petri dish containing *C. lactis* was positioned (see Wang et al. 2024 for details of the rearing set-up). The water served two functions: restricting mite movement to the plastic sheet and providing a water source. Mixed-stage *C. lactis*, along with the bran, sugar, and yeast mixture, and water, were replenished regularly. Each colony was provided with an *ad libitum* supply of prey.

All cultures and experimental units were maintained in plexiglass cabinets under constant environmental conditions: 24 °C ± 1 °C, 80% ± 5% relative humidity, and a 16:8 h (light: dark) photoperiod.

### Rearing cells

Both individual rearing and mating experiments of phytoseiid predators were conducted using modified Munger cells (see Zhang and Zhang 2021 for details). Each Munger cell consisted of two transparent plexiglass slides (38 mm × 25 mm × 2 mm). The upper plexiglass slide had a central conical hole (1 mm diameter at the top, narrowing to 0.6 mm at the base) to house the mites. The top of the hole was covered by a layer of food wrap, which was pierced with five small holes (created using a size 0 insect pin) to allow ventilation. A black plastic sheet disc (1.5 mm in diameter) was placed underneath to cover the base of the hole and provide contrast for better mite observation. A stack of filter papers below the disc served as a water reservoir, with the plastic disc pierced with five holes to allow access. The lower plexiglass slide, without apertures, was placed underneath the filter papers to maintain moisture. The two slides were secured with a pair of metal clips.

To minimise prey–predator interactions during the experiments and reduce potential mating interference, frozen feed (*C. lactis*) was prepared according to Liu et al. (2024a). The collected *C. lactis* was frozen at − 18 °C for at least 1 week and thawed at room temperature (~ 25 °C) for 30 min before being fed to the phytoseiid species during experimentation.

### Experiment 1: interspecific mating

This experiment investigated whether *A. herbicolus* females would accept mating with heterospecific males. The procedures were as follows.

1. **Rearing**: Similar-aged predator species were established by collecting nymphs (protonymphs or deutonymphs) from the main culture and rearing them individually in the rearing cells with an *ad libitum* supply of the frozen feed until maturity. Males of *Ad. limonicus*, *A. lentiginosus*, and *N. cucumeris* were used in this experiment, whereas females were used in Experiment 2.
2. **Pairing**: One newly emerged adult female (< 24 h old) of *A. herbicolus* was paired with one newly emerged male (< 24 h old) of either *Ad. limonicus*, *A. lentiginosus* or *N. cucumeris*, in a new cell with an *ad libitum* amount of the frozen feed.
3. **Observation protocol**: Mating pairs were observed every 15 min over an 8 h period under a dissection microscope to observe their interactions. Subsequently, pairs were left to mate overnight without observation, with final observations made at the 24 h mark.
4. **Endospermatophore verification**: At the end of the 24 h period, females of *A. herbicolus* were slide-mounted (Walter and Krantz 2009) to check for the presence of endospermatophores, indicating successful insemination.

### Experiment 2: intraspecific mating

This experiment evaluated whether males that had previously interacted with *A. herbicolus* females could successfully mate with conspecific females. The procedures were as follows.

1. **Pairing**: With the same set-up as in Experiment 1, adult virgin females (< 72 h old) of *Ad. limonicus*, *A. lentiginosus*, and *N. cucumeris* (obtained from the rearing procedure used in Experiment 1) were paired with males of the same species that had previously interacted with *A. herbicolus* females for 24 h during Experiment 1.
2. **Observation protocol**: Each mating pair was continuously observed under a dissection microscope from the start of the experiment until the onset of copulation (i.e., the female and male engage in the venter-to-venter position with minimal movement immediately after male oscillation). Subsequently, observations were made every 15 min until separation. Pairs where no mating was observed during the 8 h period were left to mate overnight without observation, with final checks made at the 24 h mark.
3. **Endospermatophore verification**: Post-mating, females were slide-mounted to verify endospermatophore presence as evidence of successful insemination, as in Experiment 1.

To mitigate the high propensity for intra- and inter-specific predation within the Phytoseiidae family (Gu et al. 2024; Zhang and Zhang 2022c, 2023), sufficient food (frozen mixed-stage *C. lactis*) was provided to each mating pair throughout both Experiments 1 & 2.

The mating latency (time from pairing to the onset of copulation), pre-mating behaviours, and mating durations were recorded. All examined individuals were slide-mounted, and their dorsal plate length measured using NIS-Elements (version 5.10) under a phase-contrast microscope (Eclipse 90i, Nikon Corporation, Japan). The volumes of endospermatophores were estimated using the standard equation of a sphere (Ullah et al. 2017a).

The method for recording the degree of insemination of spermathecae was adopted from Amano and Chant (1979); the first number indicates the number of inflated vesicles (without endospermatophores), while the second number indicates the number of vesicles containing endospermatophores.

### Statistical analysis

All statistical analyses were performed using R (R Core Team 2022) in RStudio (version 2023.12.1). Data visualisation was carried out with the *ggplot2* package (version 3.4.3) (Wickham 2016). Data were summarised as means with standard errors of the mean (SEMs). Dorsal plate lengths were presented in box plots, showing the interquartile range (IQR; middle 50%), median, and data spread (1.5 times the IQR). Outliers, where present, were retained unless specified otherwise. Given the non-normal distributions of all data from the Shapiro–Wilk test, the Kruskal–Wallis test and Dunn’s test were applied to determine significance. Chi-squared tests were used to compare proportions. Spearman’s rank correlations were done to determine the relationship between the volume of endospermatophores, male size, and copulation duration. Statistical significance was set at *p* < 0.05.

## Results

### Experiment 1: interspecific mating

**Fig. 1:**
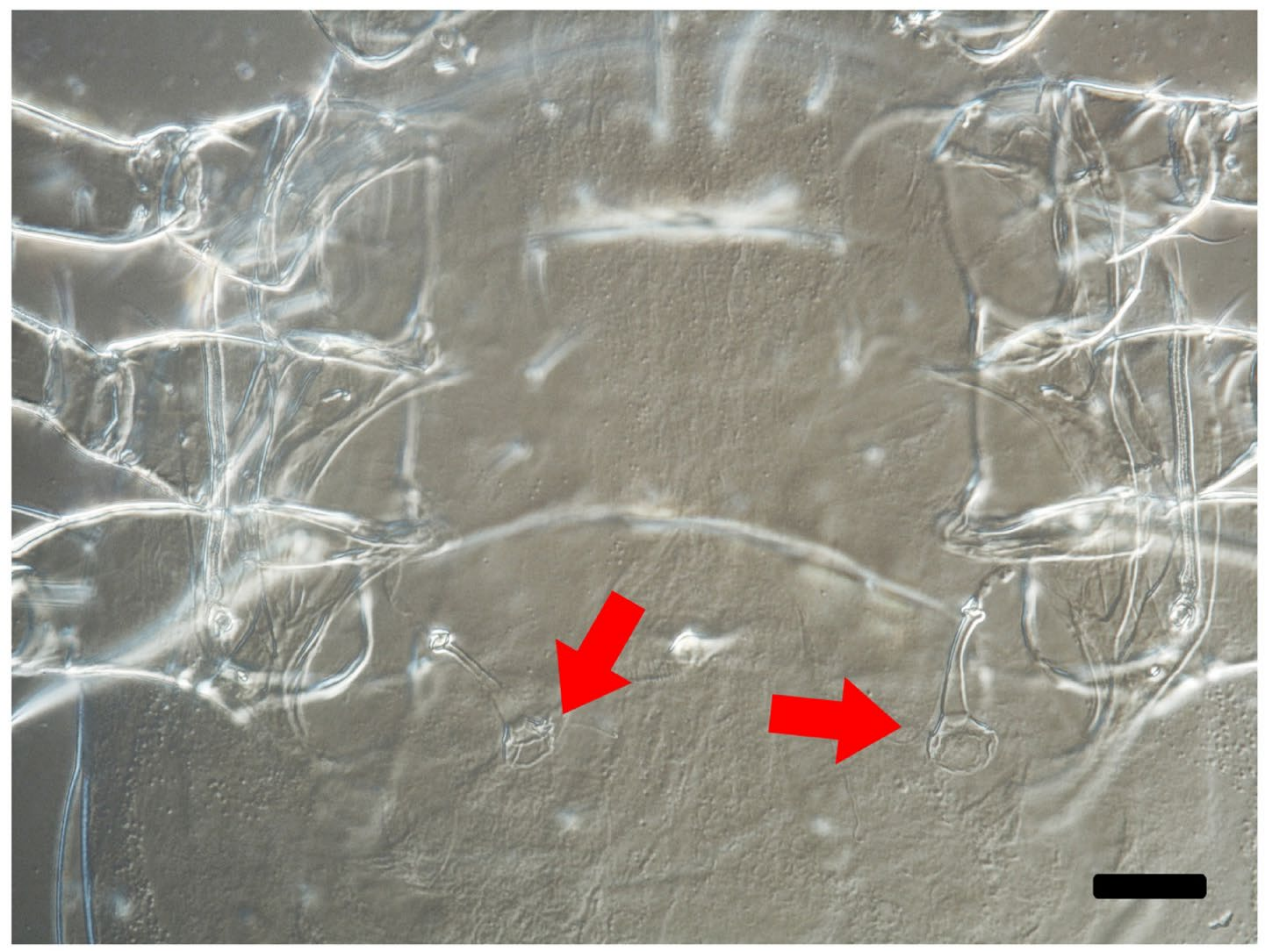
Spermathecae of *Amblyseius herbicolus* under a phase-contrast microscope at 400× magnification. Arrows point to the vesicles. Scale bar = 25 μm

No mating events were observed between *A. herbicolus* females and males of the three predator species during the first 8 h of observation. Also, slide-mounted females examined after 24 h revealed neither endospermatophores nor inflated vesicles (Table 1; Fig. 1). Occasional brief contacts were noted between *A. herbicolus* females and males of the other species, but these encounters ended without prolonged interaction. A small proportion of males were preyed upon by *A. herbicolus* females, with no significant difference in predation rates among the three species (Chi-squared test: χ² = 0.375, *df* = 2, *p* = 0.829) (Table 1).

**Table 1:** Number of mating events (first 8 h) and endospermatophores detected in *Amblyseius herbicolus* females after 24 h of exposure to males of three predator species: *Amblydromalus limonicus*, *Amblyseius lentiginosus*, and *Neoseiulus Cucumeris*

Male	*N*	Mating events	Spermatophores	Males eaten
*Ad. limonicus*	13	0	0	7.7%
*A. lentiginosus*	14	0	0	14.3%
*N. cucumeris*	11	0	0	18.2%

**Fig. 2:**
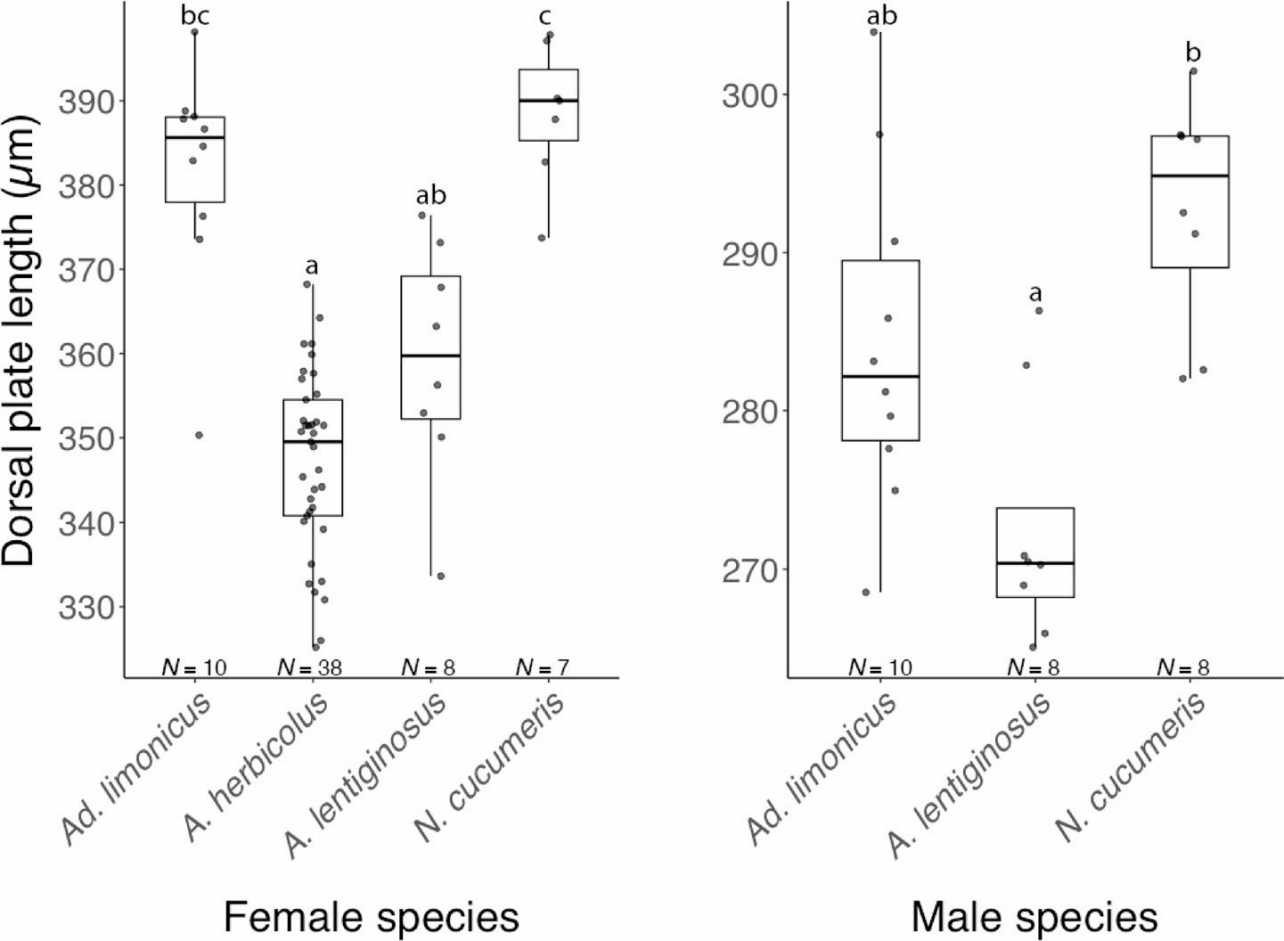
Dorsal plate length of *Amblyseius herbicolus* females, and *Amblydromalus limonicus*, *Amblyseius lentiginosus*, and *Neoseiulus cucumeris* females and males. Box plots represent interquartile range (IQR), median, and data spread (1.5× IQR). Different letters denote significant differences (Dunn’s test: *p* < 0.05). Sample sizes (*N*) are given under each box

Significant differences were found in body size (i.e., dorsal plate length) among the predator species for both females (Kruskal–Wallis test: χ² = 34.989, d*f* = 3, *p* < 0.001) and males (χ² = 10.541, *df* = 2, *p* = 0.005). Specifically, both males and females of *N. cucumeris* were the largest, while *A. herbicolus* females were similar in size to *A. lentiginosus* but approximately 10% smaller than *Ad. limonicus* and *N. cucumeris* (Fig. 2).

### Experiment 2: intraspecific mating

**Fig. 3:**
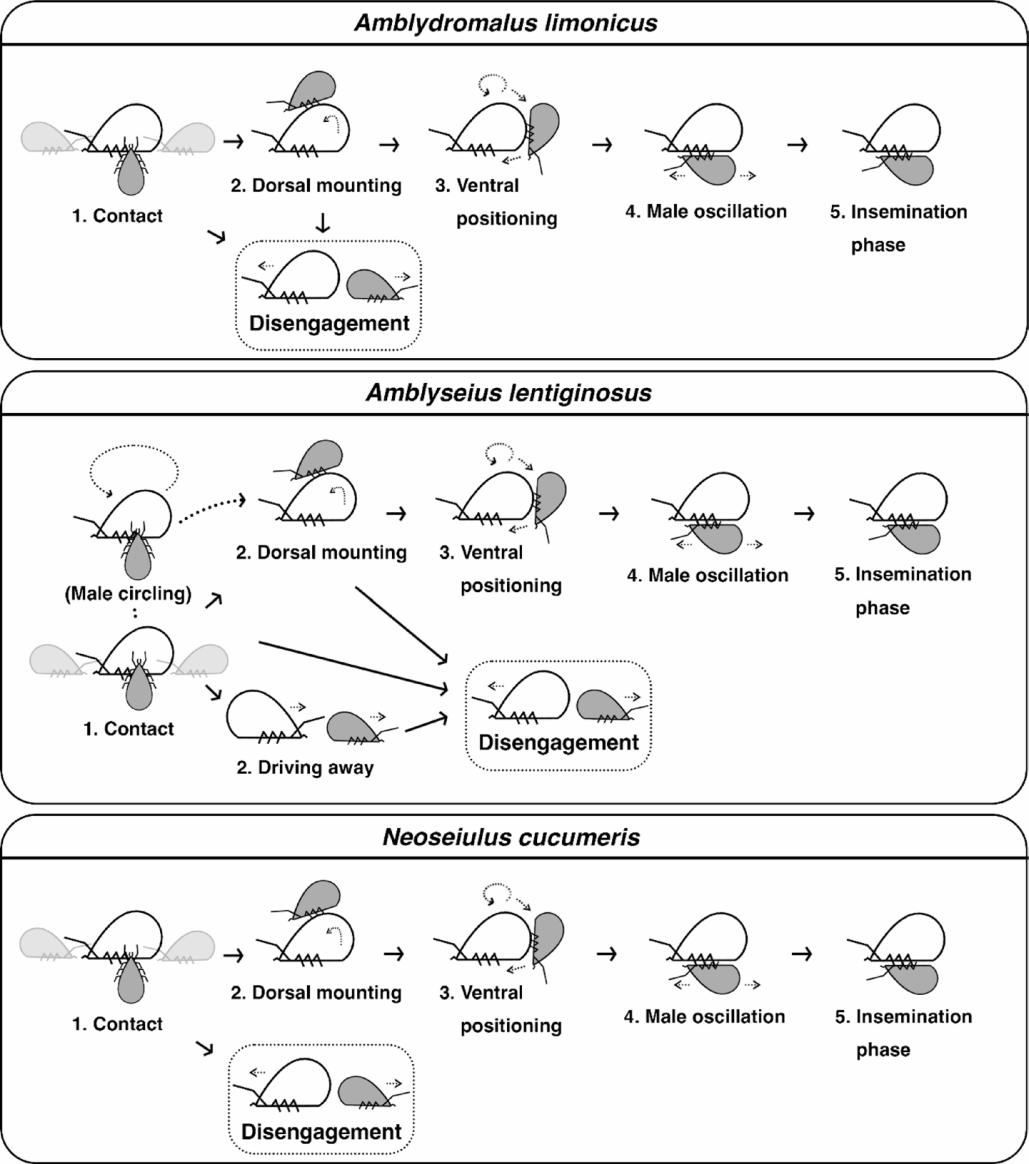
Stages of mating behaviour in *Amblydromalus limonicus*, *Amblyseius lentiginosus*, and *Neoseiulus cucumeris*

The mating process in *Ad. limonicus*, *A. lentiginosus*, and *N. cucumeris* was categorised into five sequential stages (Fig. 3), as follows.

1. **Contact**: Males initiated contact with females using their first pair of legs (Leg I). Some females also touched males with Leg I. Contact direction depended on male approach (front, behind, or side). Some males moved onto the female’s dorsum after the first contact. Only three *A. lentiginosus* males circled stationary females while touching them with Leg I before mounting.
2. **Dorsal mounting**: Males mounted the female’s dorsum, mostly from the posterior end. Some disengaged after this stage. In one *A. lentiginosus* mating pair, the female drove the male away twice after the initial contact.
3. **Ventral positioning**: Males turned around and moved to the female’s ventral side from the posterior. Females often lifted their bodies to assist males by creating more space.
4. **Male oscillation**: Males performed oscillatory (back-and-forth or sideways) movements (or jerking) before becoming stabilised for insemination.
5. **Mating or insemination phase**: Both sexes remained relatively stationary. Occasionally, females moved around with males attached to their ventrum.

**Fig. 4:**
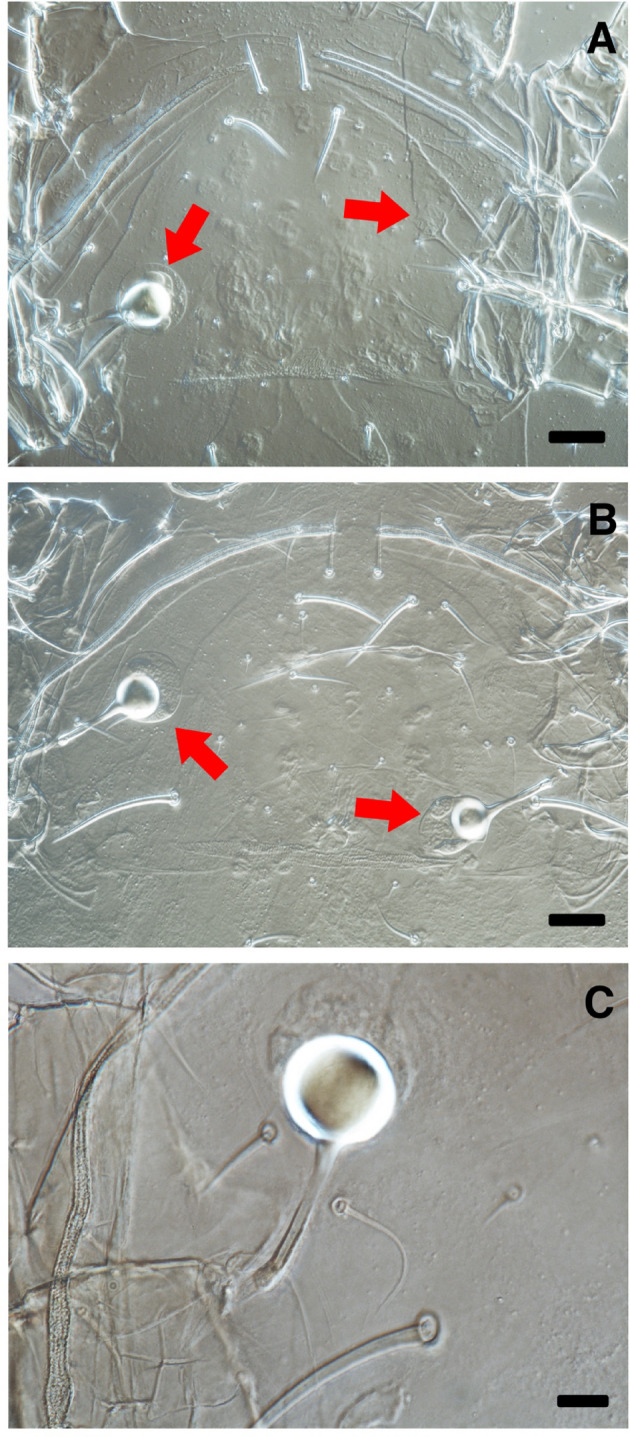
Spermathecae of *Amblydromalus limonicus* under a phase-contrast microscope. The balloon-shaped structures within the vesicles are endospermatophores. **A**: A spermatophore found in one vesicle. **B**: One spermatophore is found in each of the vesicles. **C**: An enlarged view of a vesicle. Arrows point to the vesicles. The magnifications are 400× (A & B) and 1000× (C). Scale bars = 25 μm (A & B) and 10 μm (C)

**Fig. 5:**
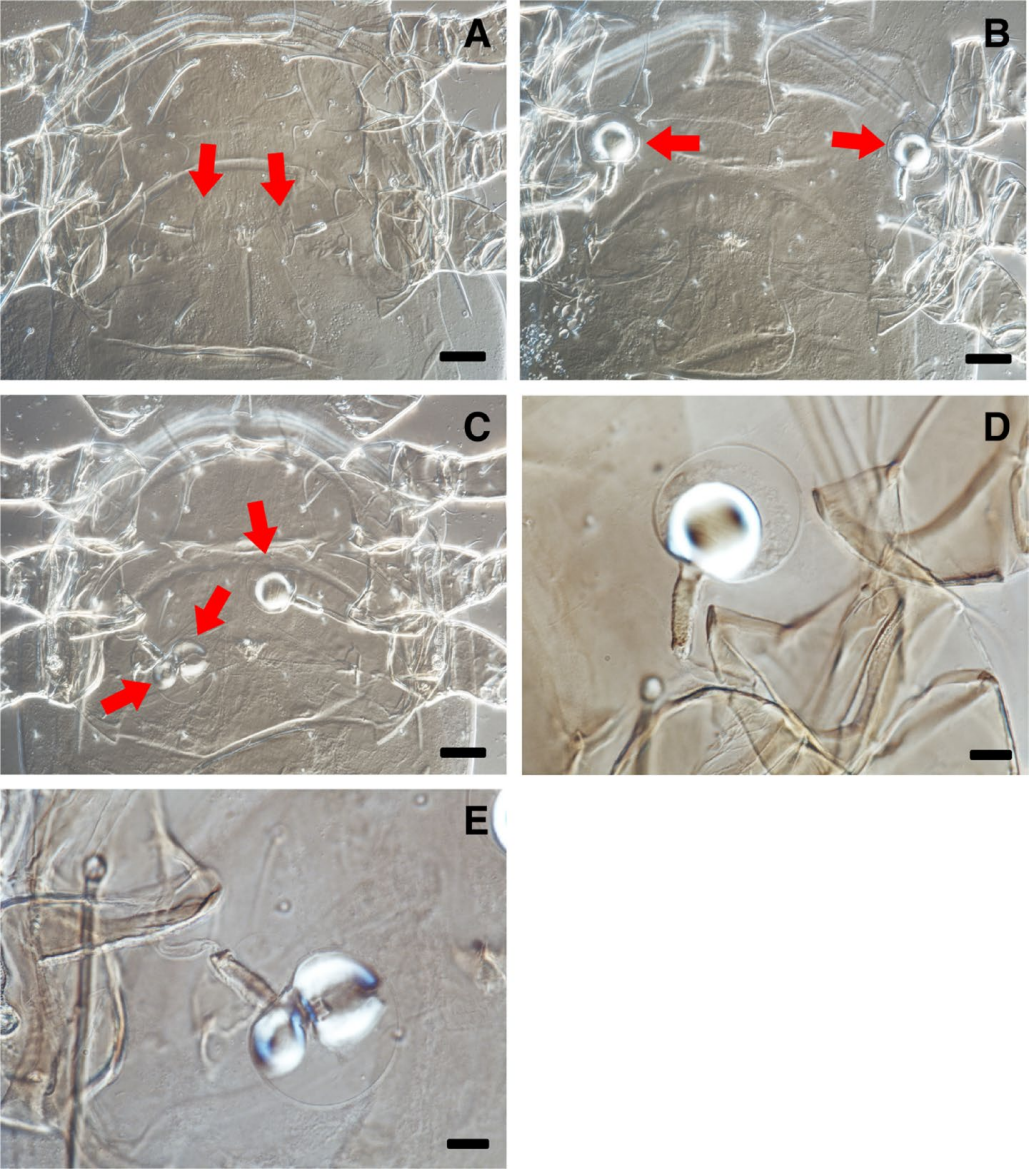
Spermathecae of *Amblydromalus lentiginosus* under a phase-contrast microscope. The balloon-shaped structures within the vesicles are endospermatophores. **A**: No spermatophores in the vesicles. **B**: Spermatophores are found in each of the vesicles. **C**: One spermatophore is found in one vesicle and two in the other. **D**: An enlarged view of a vesicle with one spermatophore. E: An enlarged view of a vesicle with two spermatophores. Arrows point to the vesicles. The magnifications are 400× (A–C) and 1000× (D & E). Scale bars = 25 μm (A–C) and 10 μm (D & E)

**Fig. 6:**
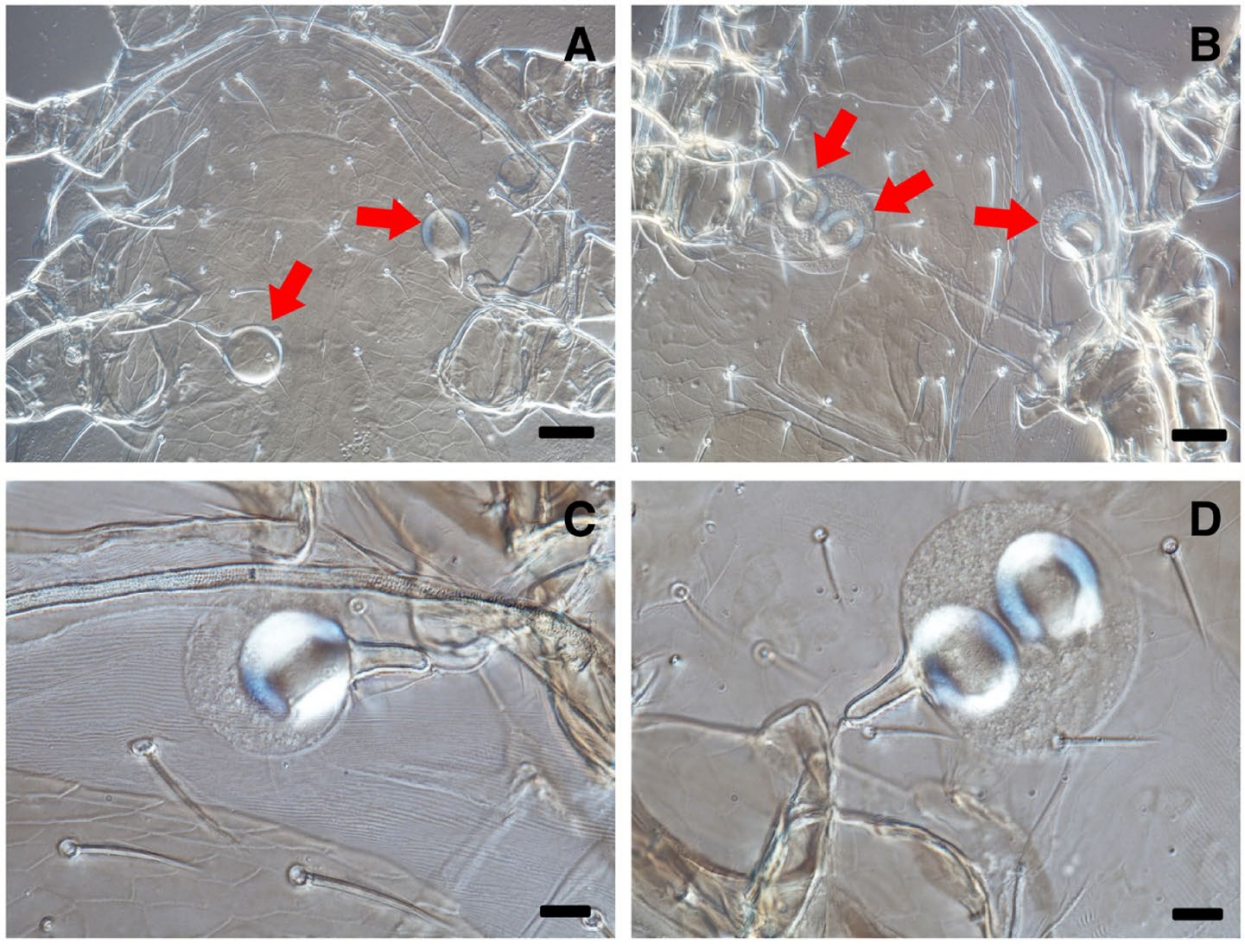
Spermathecae of *Neoseiulus cucumeris* under a phase-contrast microscope. The balloon-shaped structures within the vesicles are endospermatophores. **A**: Spermatophores are found in one of each vesicle. **B**: One spermatophore is found in one vesicle and two in the other. **C**: An enlarged view of a vesicle with one spermatophore. **D**: An enlarged view of a vesicle with two spermatophores. Arrows point to the vesicles. The magnifications are 400× (A & B) and 1000× (C & D). Scale bars = 25 μm (A & B) and 10 μm (C & D)

Endospermatophores were detected in all examined females following a single mating event, except in three *A. lentiginosus* females (Table 2; Figs. 4, 5 and 6). Two pairs of *A. lentiginosus* failed to copulate within 8 h, though one female from these pairs had endospermatophores after 24 h (Fig. 5C), suggesting delayed insemination. Also, two pairs of *A. lentiginosus* had early termination during mating (< 30 min), with no endospermatophores detected and no inflated spermathecae post-mounting. These data were excluded from further analysis of mating duration. The occurrence of mating was statistically similar among species (Chi-squared test: χ² = 4.547, *df* = 2, *p* = 0.103) (Table 2). However, successful insemination was significantly lower in *A. lentiginosus* than in the other species (χ² = 7.093, *df* = 2, *p* = 0.029).

**Table 2:** Proportion of successful mating events (0–8 h) and endospermatophores detected after a single mating event or at 24 h in *Amblydromalus limonicus*, *Amblyseius lentiginosus*, and *Neoseiulus Cucumeris*

Male	*N*	Mating events	Endospermatophores
*Ad. limonicus*	10	100%	100%
*A. lentiginosus*	9	77.8%	66.7%
*N. cucumeris*	9	100%	100%

Mating latency differed significantly among species (Kruskal–Wallis test: χ^2^ = 6.730, *df* = 2, *p* = 0.035), with *Ad. limonicus* initiating the most rapid insemination (Table 3). However, the number of initial contacts did not differ significantly across species (χ^2^ = 0.502, *df* = 2, *p* = 0.778). The duration of pre-mating behaviours was significantly longer in *A. lentiginosus* than in the other two species (χ^2^ = 10.676, *df* = 2, *p* = 0.005). This included extended durations for initial contact (χ^2^ = 8.608, *df* = 2, *p* = 0.014), dorsal mounting (χ^2^ = 9.922, *df* = 2, *p* = 0.007), and ventral positioning (χ^2^ = 10.161, *df* = 2, *p* = 0.006), whereas the duration of male oscillation did not vary significantly among species (χ^2^ = 4.399, *df* = 2, *p* = 0.111) (Table 3). Despite its prolonged pre-mating phase, *A. lentiginosus* exhibited the shortest mating duration among the three species (χ^2^ = 7.930, *df* = 2, *p* = 0.019) (Table 3).

**Table 3:** Pre-mating and mating parameters in mating of *Amblydromalus limonicus*, *Amblyseius lentiginosus*, and *Neoseiulus cucumeris*. All data are summarised as mean ± SEM

Species	*N*	Mating latency		Pre-mating duration					Mating duration (h)
		Duration (min)	Contact (number)	Contact (sec)	Mounting (sec)	Positioning (sec)	Oscillation (sec)	Total (sec)	
*Ad. limonicus*	10	5.1 ± 1.2^a^	3.4 ± 1.1	7.2 ± 1.7^a^	7.1 ± 1.4^a^	2.4 ± 0.4^a^	56.6 ± 6.3	73.3 ± 8.4^a^	5.5 ± 0.3^b^
*A. lentiginosus*	7	22.7 ± 10.0^b^	4.3 ± 1.5	36.3 ± 16.2^b^	29.5 ± 8.4^b^	9.0 ± 2.4^b^	80.8 ± 7.7	155.7 ± 29.6^b^	4.0 ± 0.3^a^*
*N. cucumeris*	9	11.7 ± 2.8^b^	2.9 ± 0.5	10.7 ± 1.6^ab^	7.4 ± 1.0^a^	3.0 ± 0.4^a^	68.3 ± 7.1	89.4 ± 6.4^a^	6.0 ± 0.6^b^

Note: Different letters denote significant differences (Dunn’s test, *p* < 0.05). * For *A. lentiginosus*, two pairs had a mating duration of < 30 min and lacked endospermatophores upon slide-mounting; these were considered early terminations and excluded from further analyses, reducing the sample size (*N*) for mating duration to five

The number of endospermatophores differed among species (Kruskal–Wallis test: χ^2^ = 6.730, *df* = 2, *p* = 0.035), with *Ad. limonicus* having fewer endospermatophores compared to *N. cucumeris* and *A. lentiginosus* (Table 4). Most females of *A. lentiginosus* and *N. cucumeris* received two or more spermatophores (typically one in each spermatheca, except for one *N. cucumeris* female that received two spermatophores in one spermatheca). In contrast, 70% of *Ad. limonicus* females had only one spermatophore, which was distributed between the left (~ 40%) and right (~ 60%) spermathecae; the side preference of male *Ad. limonicus* was not statistically significant (Binomial test: *p* = 1). Only one *Ad. limonicus* female had an inflated spermathecal vesicle without an endospermatophore (Table 4), whereas all other inflated vesicles contained at least one endospermatophore.

**Table 4:** Number, degree of insemination, and size (individual volume) of endospermatophore after one mating event of *Amblydromalus limonicus*, *Amblyseius lentiginosus*, and *Neoseiulus cucumeris*. Means ± SEM are given for the number and size of endospermatophores

Species	*N*	Number	Degree of insemination					*N*	Size (mm^3^)
			0–0	1–0	1–1	2–1	2–2		
*Ad. limonicus*	10	1.3 ± 0.2 (1–2)^a^	0	0	6	1	3	13	4.4 × 10^−5^ ± 2.1 × 10^−6 a^
*A. lentiginosus*	5	2.0 ± 0.0 (2)^b^	0	0	0	0	5	13	4.8 × 10^−5^ ± 3.5 × 10^−6 ab^
*N. cucumeris*	7	2.1 ± 0.1 (2–3)^b^	0	0	0	0	7	15	6.2 × 10^−5^ ± 5.0 × 10^−6 b^

Different letters denote significant differences (Dunn’s test, *p* < 0.05). Degree of insemination: number of inflated vesicles minus number of inseminated vesicles

**Fig. 7:**
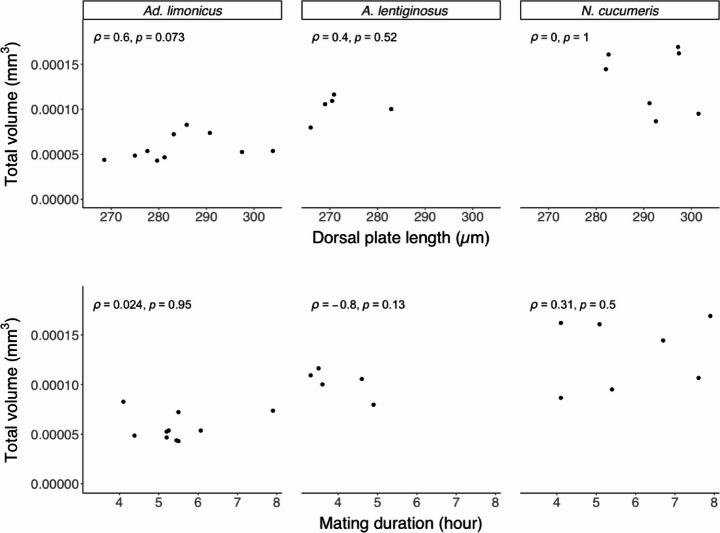
Correlation between individual male size, mating duration, and the total volume of endospermatophores inseminated into females’ spermathecae of *Amblydromalus limonicus*, *Amblyseius lentiginosus*, and *Neoseiulus cucumeris* by each conspecific male during a single mating event. Spearman’s rank correlation coefficients () and *p*-values are given in each graph

Spermatophore size also varied significantly among species (χ² = 9.354, *df* = 2, *p* = 0.009), with *N. cucumeris* producing the largest spermatophores, whereas those of *A. lentiginosus* were comparable to both *N. cucumeris* and *Ad. limonicus* (Table 4). However, there were no significant correlations between male body size, mating duration, and total volume of spermatophores for any of the three species (Fig. 7).

## Discussion

In this study mating and insemination between *A. herbicolus* females and males of *Ad. limonicus*, *A. lentiginosus*, and *N. cucumeris* were not observed (Experiment 1). However, all males, except three *A. lentiginosus* individuals, successfully inseminated a conspecific female when exposed to one (Experiment 2). These findings indicate that interspecific mating between *A. herbicolus* females and males of the other three species is improbable. In addition to differences in body size, these species exhibit distinct morphological characteristics (Zhang 2003).

It is possible that *A. herbicolus* females and the heterospecific males tested in this study might have been able to distinguish one another, as species recognition has been documented in members of Phytoseiidae, including *N. cucumeris* (Schausberger and Croft 2000). Moreover, some males of all three species examined served as intraguild prey for *A. herbicolus*, with no apparent predation preference among male species. However, the absence of mating behaviour does not necessarily indicate that *A. herbicolus* females are incapable of mating, either behaviourally or physiologically. Future studies could investigate their compatibility with *Amblyseius largoensis* (Muma), a more closely related species with greater morphological and genetic similarities to *A. herbicolus* (Ma et al. 2019; dos Santos and Tixier 2017).

The courtship sequences of conspecific pairs of *Ad. limonicus*, *A. lentiginosus*, and *N. cucumeris* were similar and conformed to the *Amblyseius*-*Typhlodromus* type described by Amano and Chant (1979). Since phytoseiid species lack functional eyes (Liu et al. 2024b), whether the mating sequence functions as a courtship display or a form of pre-mating stimulation requires further investigation. However, significant interspecific differences were observed in the duration of pre-mating and mating stages, which suggest possible species-specific adaptations. Whether these differences arise from variations in mate recognition, mate choice or courtship behaviour remains unclear. In addition to the longest pre-mating duration among examined species, *A. lentiginosus* exhibited a lower insemination rate than *Ad. limonicus* and *N. cucumeris*, with two incidents of early mating termination. Also, one *A. lentiginosus* female was observed chasing the male away after contact. These results suggest that *A. lentiginosus* females might be more selective.

The number and size of spermatophores inseminated after the first mating event further highlight interspecific differences in reproductive strategies (e.g., sperm allocation). Males of *Ad. limonicus* were similar to those of the phytoseiid predators *P. persimilis* and *Kampimodromus aberrans*, mostly inseminating one spermatophore to one of the spermathecae (Amano and Chant 1979; Pappas et al. 2005). In contrast, males of *A. lentiginosus* and *N. cucumeris* showed a pattern comparable to *Amblyseius andersoni*, typically inseminating two spermatophores, one into each spermatheca (Amano and Chant 1979).

The different numbers and sizes of endospermatophores between species may reflect a different level of sperm allocation to mating, or adaptations to maximise fertilisation success and male reproductive fitness. For example, in *P. persimilis*, which exhibits low levels of polyandry and where females often avoiding multiple matings (Amano and Chant 1978; Schausberger et al. 2017), males may not need to transfer excess sperm to ensure reproductive success. Conversely, in *N. cucumeris*, where polyandry is more common (Ji et al. 2007), males may benefit from inseminating both spermathecae to enhance their reproductive success. However, this hypothesis requires further investigation, particularly to determine the level of polyandry in *Ad. limonicus* and *A. lentiginosus*.

**Fig. 8:**
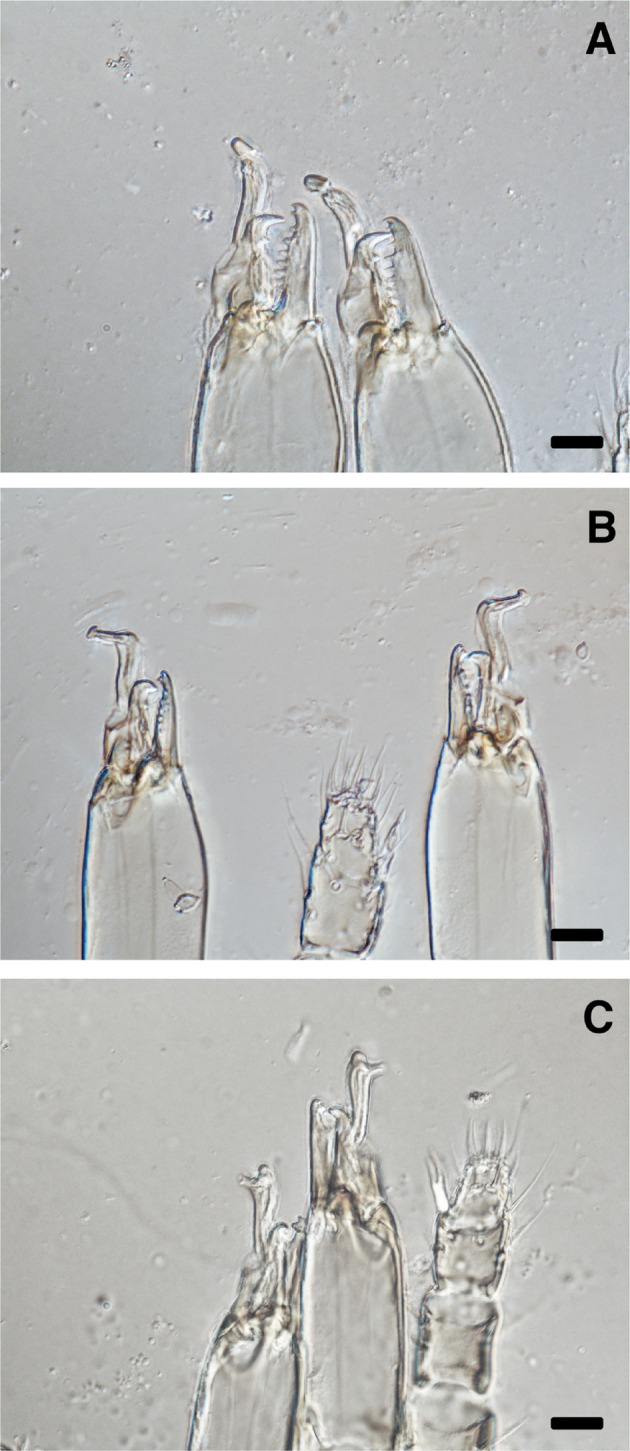
Chelicerae of the examined phytoseiid male species in this study, showing the spermatodactyl structure on the movable digits at 1000× magnification. **A**: *Amblydromalus limonicus*; **B**: *Amblyseius lentiginosus*; and **C**: *Neoseiulus cucumeris*. Scale bar = 10 μm

There are morphological differences in the structure of the spermatodactyl on the movable digit of the male chelicerae (Fig. 8). Whether these structural differences relate to their mating behaviour and degree of insemination requires further investigation.

No correlation was found between male body size, mating duration, and total volume of endospermatophores after the first mating in any species examined. A positive relationship between mating duration and endospermatophore size has been reported in several phytoseiid species, including *Neoseiulus womersleyi*, *Neoseiulus longispinosus*, and *P. persimilis* (Schulten et al. 1978; Ullah et al. 2017a). The absence of such a correlation in this study may be due to all individuals being allowed to complete the full mating sequence or to the limited sample size. Future studies incorporating larger sample sizes may reveal more informative patterns. Also, investigating whether male body size influences lifetime sperm production, rather than sperm allocation per mating, could provide further insight into the selective advantages of larger body size beyond increased female receptivity and competitive ability (Schausberger et al. 2017).

One limitation of this study was that the prolonged confinement of *A. herbicolus* females with heterospecific males in enclosed arenas may have influenced subsequent mating behaviour with conspecifics. Females of *A. herbicolus*, as superior intraguild predators, may have induced stress in the males, subsequently affecting their physiological and behavioural responses (Gu et al. 2022, 2024). Furthermore, forced pairings in artificial conditions may not accurately reflect natural mating dynamics. The multiple contacts observed between pairs before insemination suggest a tendency to seek alternative mates. Future research on phytoseiid mating behaviour would benefit from more ecologically relevant experimental designs that incorporate natural choice conditions.

In conclusion, *A. herbicolus* females did not accept mating from heterospecific males of *Ad. limonicus*, *A. lentiginosus*, and *N. cucumeris*. Further research should investigate the mechanisms underlying and maintaining parthenogenesis in this species, such as the potential role of endosymbionts such as *Wolbachia*, which induce parthenogenesis in other insects and mites (Ramalho et al. 2021; Weeks and Breeuwer 2001). Also, the observed differences in pre-mating duration, mating behaviours, and insemination strategies among *Ad. limonicus*, *A. lentiginosus*, and *N. cucumeris* may reflect adaptations to distinct ecological pressures, including mate availability, predation risk, and sperm competition.

## Electronic Supplementary Material

Below is the link to the electronic supplementary material.

Supplementary Material 1

## Data Availability

The original data will be deposited in an open-access database once the paper is accepted. A link to DOI of the dataset will be added to the paper before publication.

## References

[CR1] Alberti G (2002) Ultrastructural investigations of sperm and genital systems in Gamasida (Acari: Anactinotrichida): current state and perspectives for future research. Acarologia 42:107–126

[CR2] Amano H, Chant DA (1977) Life history and reproduction of two species of predacious mites, *Phytoseiulus persimilis* Athias-Henriot and *Amblyseius andersoni* (Chant) (Acarina: Phytoseiidae). Can J Zool 55:1978–1983. 10.1139/z77-255

[CR3] Amano H, Chant DA (1978) Some factors affecting reproduction and sex ratios in two species of predacious mites, *Phytoseiulus persimilis* Athias-Henriot and *Amblyseius andersoni* (Chant) (Acarina: Phytoseiidae). Can J Zool 56:1593–1607. 10.1139/z78-221

[CR4] Amano H, Chant DA (1979) Mating behaviour and reproductive mechanisms of two species of predacious mites, *Phytoseiulus persimilis* Athias-Henriot and *Amblyseius andersoni* (Chant) (Acarina: Phytoseiidae). Acarologia 20:196–213

[CR5] Cavalcante ACC, dos Santos VLV, Rossi LC, de Moraes GJ (2015) Potential of five Brazilian populations of Phytoseiidae (Acari) for the biological control of *Bemisia tabaci* (Insecta: Hemiptera). J Econ Entomol 108:29–33. 10.1093/jee/tou003 26470100 10.1093/jee/tou003

[CR6] Cavalcante ACC, Demite PR, Amaral FSR, Lofego AC, de Moraes GJ (2017) Complementary description of *Neoseiulus Tunus* (De Leon) (Acari: mesostigmata: Phytoseiidae) and observation on its reproductive strategy. Acarologia 57:591–599. 10.24349/acarologia/20174178

[CR7] dos Santos VV, Tixier M (2017) Which molecular markers for assessing which taxonomic level? The case study of the mite family Phytoseiidae (Acari: Mesostigmata). Cladistics 33:251–267. 10.1111/cla.12166 34715727 10.1111/cla.12166

[CR8] Elbadry EA, Elbenhawy EM (1968) Studies on the mating behaviour of the predaceous mite *Amblyseius Gossipi* [Acarina, Phytoseiidae]. Entomophaga 13:159–162. 10.1007/bf02371786

[CR9] Fan Q, Ma M, Zhang Z (2024) New records of Phytoseiidae (Acari: Mesostigmata) in new zealand, with notes on the occurrence status of some species. Syst Appl Acarol 29:200–213. 10.11158/saa.29.2.2

[CR10] Fang X, Li J, Wu W (2022) Phytoseiid mites of Ruyuan Yao autonomous county, China (Acari: mesostigmata, Phytoseiidae). Acarologia 62:474–496. 10.24349/l0py-4r2b

[CR11] Girard MB, Kasumovic MM, Elias DO (2011) Multi-modal courtship in the Peacock spider, *Maratus volans* (O.P.-Cambridge, 1874). PLoS ONE 6:e25390–e25390. 10.1371/journal.pone.0025390 21980440 10.1371/journal.pone.0025390 PMC3181266

[CR12] Gu X, Zhang K, Zhang Z (2022) Non-consumptive effects of intraguild predator *Blattisocius dentriticus* (Berlese) on the development and prey consumption of *Neoseiulus Cucumeris* (Oudemans). Syst Appl Acarol 27:1475–1482. 10.11158/saa.27.7.12

[CR13] Gu X, Zhang K, Zhang Z (2024) Interspecific interactions between two predatory mites of the mould mite *Tyrophagus putrescentiae* Schrank (Acari: Acaridae), a serious pest of stored products. J Stored Prod Res 105:102255. 10.1016/j.jspr.2024.102255

[CR14] Hou F, Ni Z, Zou M, Zhu R, Yi T, Guo J, Jin D (2022) The effects of alternative foods on life history and cannibalism of *Amblyseius herbicolus* (Acari: Phytoseiidae). Insects 13:1036. 10.3390/insects13111036 36354860 10.3390/insects13111036 PMC9699404

[CR15] Hoy MA (1985) Recent advances in genetics and genetic improvement of the Phytoseiidae. Annu Rev Entomol 30:345–370. 10.1146/annurev.en.30.010185.002021

[CR16] Hoy MA, Cave FE (1985) Mating behavior in four strains of *Metaseiulus occidentalis* (Acari: Phttoseiidae). Ann Entomol Soc Am 78:588–593. 10.1093/aesa/78.5.588

[CR17] Ji J, Zhang Z, Zhang Y, Chen X, Lin J (2007) Effects of mating rates on oviposition, sex ratio and longevity in a predatory mite *Neoseiulus Cucumeris* (Acari: Phytoseiidae). Exp Appl Acarol 43:171–180. 10.1007/s10493-007-9114-x 17968663 10.1007/s10493-007-9114-x

[CR18] Kalile MO, Cardoso AC, Pallini A, Fonseca MM, Elliot SL, Fialho VS, da Carvalho S, Janssen T A (2021) A predatory mite as potential biological control agent of *Diaphorina citri*. Biocontrol 66:237–248. 10.1007/s10526-020-10061-8

[CR19] Lam W, Paynter Q, Zhang Z (2021) Functional response of *Amblyseius herbicolus* (Acari: Phytoseiidae) on *Sericothrips staphylinus* (Thysanoptera: Thripidae), an ineffective biocontrol agent of gorse. Biol Control 152:104468. 10.1016/j.biocontrol.2020.104468

[CR20] Liu W, Zhang K, Zhang Z (2023) Larval feeding types shape the predation aggression of predatory mites in both intraspecific and interspecific encounters. Syst Appl Acarol 28:1272–1282. 10.11158/saa.28.7.6

[CR21] Liu Z, Zhang K, Zhang Z (2024a) Enhancing the efficiency of egg collection of the astigmatid mite *Carpoglyphus lactis* (Acari: Carpoglyphidae) as a diet for predatory mites. Syst Appl Acarol 29:355–358. 10.11158/saa.29.2.14

[CR22] Liu Z, Zhang K, Zhang Z (2024b) Phototactic behavior and oviposition of seven species of Phytoseiidae (Acari: Mesostigmata). 10.1002/ps.8575. Pest Manag Sci n/a 10.1002/ps.8575 39632773

[CR23] Liu Z, Zhang K, Zhang Z (2024c) Unintended consequences: the adverse effects of Royal jelly supplementation in the predatory mite *Amblyseius herbicolus* chant (Acari: Phytoseiidae). Syst Appl Acarol 29:335–345. 10.11158/saa.29.2.12

[CR24] Ma M, Fan Q, Zhang Z (2019) Amblyseiinae of new Zealand (Acari: Phytoseiidae): redescriptions, rediscoveries, new records, new combinations and keys to species. Zootaxa 4658:201–222. 10.11646/zootaxa.4658.2.1 10.11646/zootaxa.4658.2.1 31716741

[CR25] Ma M, Zhang K, Fan Q, Zhang Z (2024) Description of ontogenetic changes in the morphology of *Amblyseius herbicolus* (Chant, 1959) and *Amblyseius lentiginosus* Denmark & schicha, 1974 (Acari: Phytoseiidae). Zootaxa 5485:7–37. 10.11646/zootaxa.5485.1.4 39646004 10.11646/zootaxa.5485.1.4

[CR26] McMurtry JA, Moraes GJD, Sourassou NF (2013) Revision of the lifestyles of phytoseiid mites (Acari: Phytoseiidae) and implications for biological control strategies. Syst Appl Acarol 297–320. 10.11158/saa.18.4.1

[CR27] McMurtry JA, Sourassou NF, Demite PR (2015) The Phytoseiidae (Acari: Mesostigmata) as biological control agents. In: Carrillo D, de Moraes GJ, Peña JE (eds) Prospects for biological control of plant feeding mites and other harmful organisms. Springer International Publishing, Cham, pp 133–149

[CR28] Momen FM (1997) Copulation, egg production and sex ratio in *Cydnodromella negevi* and *Typhlodromus athiasae*. Phytoseiidae) Anz Schädl Kd Pflanzenschutz Umweltschutz 70:34–36. 10.1007/BF01991955. Acari

[CR29] Moraza M, Linquist EE (2011) A new genus of fungus-inhabiting blattisociid mites (Acari: mesostigmata: Phytoseioidea) from middle america, with a key to genera and subgenera of the subfamily blattisociinae. Zootaxa 2758:1–25. 10.11646/zootaxa.2758.1.1

[CR30] Norton RA, Kethley JB, Johnston DE, O’Connor BM (1993) Phylogenetic perspectives on genetic systems and reproductive modes of mites. In: Wrensch DL, Ebbert MA (eds) Evolution and diversity of sex ratio in insects and mites. Chapman & Hall, New York, pp 8–99

[CR31] Notghi Moghadam BA, Rafati-fard M, Jalali Sendi J, Hajizadeh J (2010) Influence of three diets on development and oviposition of the predatory mite, *Amblyseius herbicolus* (Acari: Phytoseiidae) under laboratory conditions. J Entomol Soc Iran 30:51–68

[CR32] Oliver JH (1971) Parthenogenesis in mites and ticks (Arachnida: Acari). Am Zool 11:283–299. 10.1093/icb/11.2.283

[CR33] Pappas ML, Broufas GD, Koveos DS (2005) Mating behavior of the predatory mite *Kampimodromus aberrans* (Acari: Phytoseiidae). Exp Appl Acarol 36:187–197. 10.1007/s10493-005-5303-7 16132733 10.1007/s10493-005-5303-7

[CR34] R Core Team (2022) R: A Language and environment for statistical computing. R Foundation Statistical Computing, Vienna. http://www.R-project.org/

[CR35] Ramalho MO, Kim Z, Wang S, Moreau CS (2021) *Wolbachia* across social insects: patterns and implications. Ann Entomol Soc Am 114:206–218. 10.1093/aesa/saaa053

[CR36] Reis PR, Teodoro AV, Pedro Neto M, da Silva EA (2007) Life history of *Amblyseius herbicolus* (Chant) (Acari: Phytoseiidae) on coffee plants. Neotrop Entomol 36:282–287. 10.1590/S1519-566X2007000200016 17607463 10.1590/s1519-566x2007000200016

[CR37] Reyer H (2008) Mating with the wrong species can be right. Trends Ecol Evol 23:289–292. 10.1016/j.tree.2008.03.001 18440091 10.1016/j.tree.2008.03.001

[CR38] Sato Y, Staudacher H, Sabelis MW (2016) Why do males choose heterospecific females in the red spider mite? Exp Appl Acarol 68:21–31. 10.1007/s10493-015-9985-1 26530994 10.1007/s10493-015-9985-1

[CR39] Schausberger P, Croft BA (2000) Cannibalism and intraguild predation among phytoseiid mites: are aggressiveness and prey preference related to diet specialization? Exp Appl Acarol 24:709–725. 10.1023/A:1010747208519 11227828 10.1023/a:1010747208519

[CR40] Schausberger P, Walzer A, Murata Y, Osakabe M (2017) Low level of polyandry constrains phenotypic plasticity of male body size in mites. PLoS ONE 12:e0188924. 10.1371/journal.pone.0188924 29190832 10.1371/journal.pone.0188924 PMC5708631

[CR41] Schulten GGM (1985) Mating. In: Helle W, Sabelis MW (eds) Spider mites: their biology, natural enemies and control, vol 1B. Elsevier Science, Amsterdam, pp 55–65

[CR42] Schulten GGM, Arendonk RCM, van Russell VM, Roorda FA (1978) Copulation, egg production and sex-ratio in phytoseiulus persimilis and Amblyseius Bibens (Acari: Phytoseiidae). Entomol Exp Appl 24:145–153. 10.1111/j.1570-7458.1978.tb02764.x

[CR43] Tsunoda T (1994) Mating behavior of the predacious mite, *Amblyseius womersleyi* schicha (Acari: Phytoseiidae). Appl Entomol Zool 29:141–147. 10.1303/aez.29.141

[CR44] Tsunoda T, Amano H (2001) Female mate-receptivity behavior in multiple matings of a predacious mite, *Amblyseius womersleyi* schicha (Acari: Phytoseiidae). Appl Entomol Zool 36:393–397. 10.1303/aez.2001.393

[CR45] Ullah MS, Sugimoto R, Kongchuensin M, Konvipasruang P, Gotoh T (2017a) Copulation duration, sperm transfer and reproduction of the two closely related phytoseiid mites, *Neoseiulus womersleyi* and *Neoseiulus longispinosus* (Acari: Phytoseiidae). Exp Appl Acarol 71:47–61. 10.1007/s10493-016-0101-y 27943023 10.1007/s10493-016-0101-y

[CR46] Ullah MS, Sugimoto R, Matsuda T, Wang C-, Kongchuensin M, Konvipasruang P, Gotoh T (2017b) Interspecific interference in the closely related predatory mites *Neoseiulus womersleyi* and *Neoseiulus longispinosus* (Acari: Phytoseiidae). Int J Acarol 43:296–301. 10.1080/01647954.2017.1293732

[CR47] Walter DE, Krantz GW (2009) Collecting, rearing, and Preparing specimens. In: Krantz GW, Walter DE (eds) A manual of acarology. Texas Tech University, Texas, pp 83–103

[CR48] Wang J, Zhang K, Li L, Zhang Z (2024) Development and reproduction of four predatory mites (Parasitiformes: Phytoseiidae) feeding on the spider mites *Tetranychus evansi* and *T. urticae* (Trombidiformes: Tetranychidae) and the dried fruit mite *Carpoglyphus lactis* (Sarcoptiformes: Carpoglyphidae). Syst Appl Acarol 29:269–284. 10.11158/saa.29.2.7

[CR49] Weeks AR, Breeuwer JA (2001) Wolbachia-induced parthenogenesis in a genus of phytophagous mites. Proc Biol Sci 268:2245–2251. 10.1098/rspb.2001.1797 11674872 10.1098/rspb.2001.1797 PMC1088872

[CR50] Wickham H (2016) Ggplot2: elegant graphics for data analysis. Springer-, New York

[CR51] Xie L, Yan Y, Zhang Z (2018) Development, survival and reproduction of *Stratiolaelaps scimitus* (Acari: Laelapidae) on four diets. Syst Appl Acarol 23:779–794. 10.11158/saa.23.4.16

[CR52] Xin T, Zhang Z (2021) Suitability of pollen as an alternative food source for different developmental stages of *Amblyseius herbicolus* (Chant) (Acari: Phytoseiidae) to facilitate predation on whitefly eggs. Acarologia 61:790–801. 10.24349/bIV1-2heN

[CR53] Xu Y, Zhang K, Han X, Zhang Z (2024) Early life food intake modulates effects of diet restriction on lifespan and fecundity in later life in a predatory mite (Acari: Phytoseiidae). 10.1093/cz/zoae047. Curr Zool:zoae047 10.1093/cz/zoae047 PMC12227415 40620589

[CR54] Zhang Z (1999) Biology and ecology of trombidiid mites (Acari: Trombidioidea). Ecology and evolution of the Acari. Springer, Dordrecht, Netherlands, pp 277–289

[CR55] Zhang Z (2003) Phytoseiid mites. Mites of greenhouses: identification, biology and control. CABI Publishing, Wallingford, UK, pp 171–179

[CR56] Zhang K (2022) Kin recognition in a predatory mite *Amblyseius herbicolus* Chant (Acari: Phytoseiidae). Dissertation, University of Auckland

[CR57] Zhang K, Zhang Z (2021) The dried fruit mite Carpoglyphus lactis (Acari: Carpoglyphidae) is a suitable alternative prey for Amblyseius herbicolus (Acari: Phytoseiidae). Syst Appl Acarol 26:2167–2176. 10.11158/saa.26.11.15

[CR58] Zhang K, Zhang Z (2022a) *Amblyseius herbicolus* mothers prefer to oviposit near eggs of non-kin in the absence of prey *Carpoglyphus lactis* (Acari: phytoseiidae, Carpoglyphidae). Syst Appl Acarol 27:2347–2354. 10.11158/saa.27.11.16

[CR59] Zhang K, Zhang Z (2022b) Kin recognition by cannibals is modulated by hunger level in a generalist predatory mite *Amblyseius herbicolus* (Chant) (Acari: Phytoseiidae). J Appl Entomol 146:579–585. 10.1111/jen.12973

[CR60] Zhang K, Zhang Z (2022c) Social context during ontogeny affects cannibalism and kin recognition of the predatory mite *Amblyseius herbicolus* at different life stages. Exp Appl Acarol 88:317–328. 10.1007/s10493-022-00764-1 36434489 10.1007/s10493-022-00764-1

[CR61] Zhang K, Zhang Z (2023) A thelytokous predatory mite is more cannibalistic towards distant kin. Curr Zool 69:578–584. 10.1093/cz/zoac074 37637319 10.1093/cz/zoac074 PMC10449414

[CR62] Zhang K, Liu Z, Zhang Z (2024) Older mothers produce smaller eggs without compromising offspring quality: A study of a thelytokous mite predator (Acari: Phytoseiidae). Bull Entomol Res 114:820–827. 10.1017/S0007485324000658 39555574 10.1017/S0007485324000658

