# Supplementary material for: Investigating interspecific mating in the thelytokous predatory mite Amblyseius herbicolus (Chant) (Acari: Phytoseiidae), with comparative observations from three sexually reproducing phytoseiid species
Source: Exp Appl Acarol. 2025 Jun 3;95(1):8. doi: 10.1007/s10493-025-01034-6 (PMC12133926; doi:10.1007/s10493-025-01034-6)

**Supplementary Information 1.** Images of adult females and males of *Amblydromalus limonicus*, *Amblyseius lentiginosus*, and *Neoseiulus cucumeris*, as well as adult females of *Amblyseius herbicolus*, captured using a camera equipped with an adapted lens at 20× magnification. Specimens were euthanised by submersion in 75% ethanol prior to imaging. Note the variation in body colouration among species.


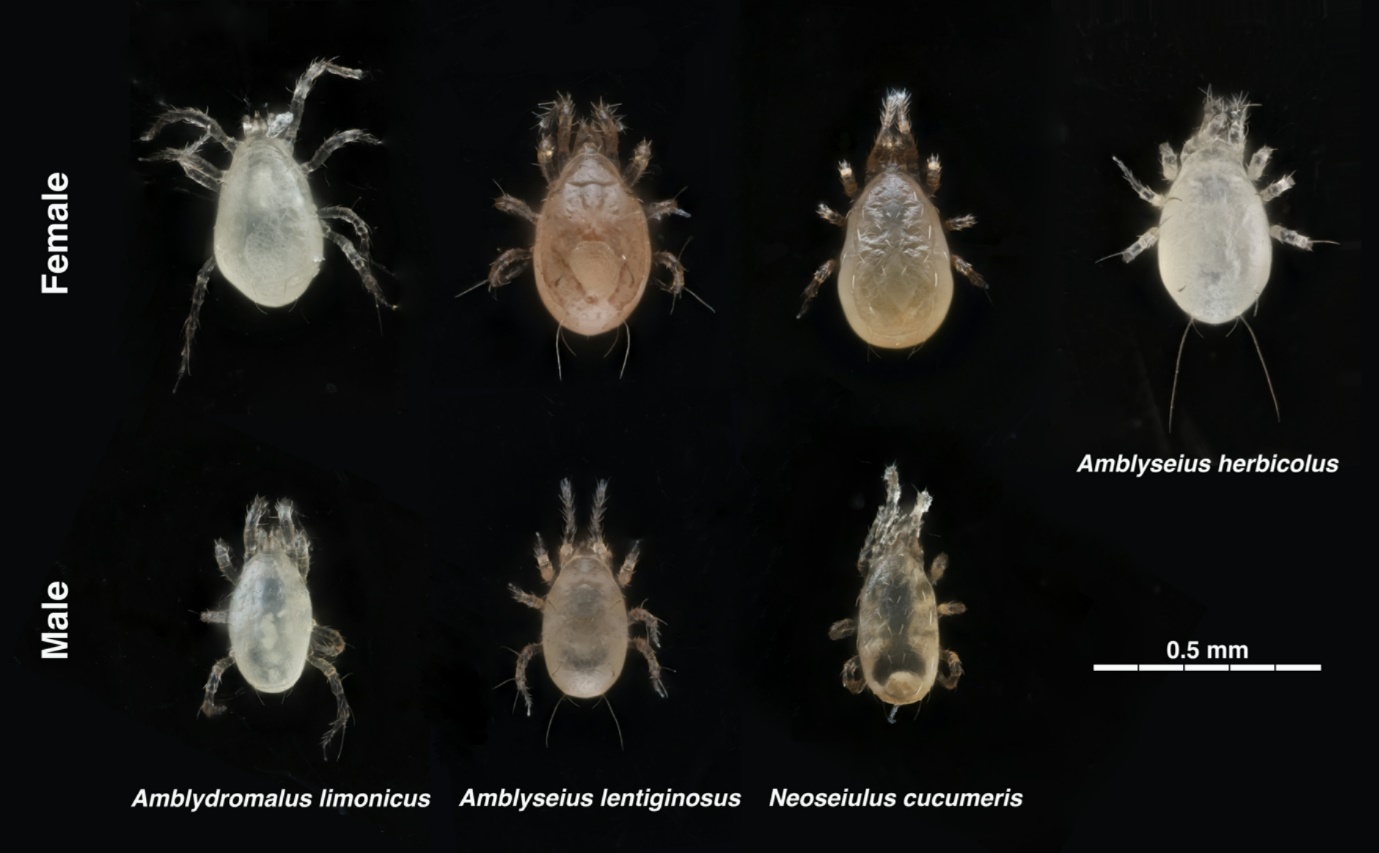


**Supplementary Information 2.** Images of mating pairs of *Amblydromalus limonicus*, *Amblyseius lentiginosus*, and *Neoseiulus cucumeris*, with females positioned ventral side up and males dorsal side up (venter-to-venter position). Images were captured using a camera with an adapted lens at 20× magnification. Specimens were euthanised by submersion in 75% ethanol prior to imaging. As a result, the positioning of the pairs may be slightly altered (males appear misaligned due to preservation) compared to *in situ* observations.


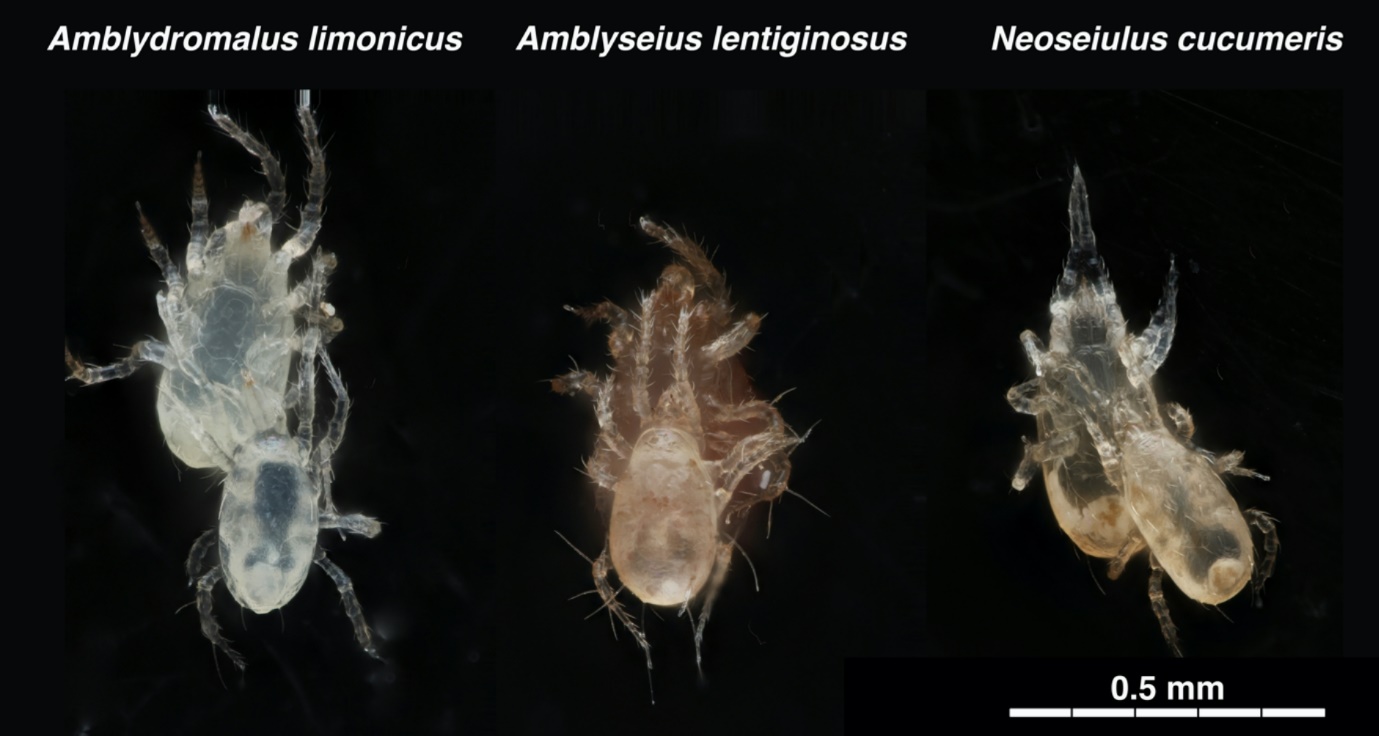

Supplement: Supplementary file 1 — Supplementary Material 1 [file 10493_2025_1034_MOESM1_ESM.docx]
